# Gastroesophageal Reflux and Idiopathic Pulmonary Fibrosis: A Review

**DOI:** 10.1155/2011/634613

**Published:** 2010-12-09

**Authors:** Ahmed Fahim, Michael Crooks, Simon P. Hart

**Affiliations:** ^1^Division of Cardiovascular and Respiratory Studies, Castle Hill Hospital, Castle Road, Cottingham HU16 5JQ, UK; ^2^Division of Cardiovascular and Respiratory Studies, Hull York Medical School, Castle Hill Hospital, Castle Road, Cottingham HU16 5JQ, UK

## Abstract

The histological counterpart of idiopathic pulmonary fibrosis is usual interstitial pneumonia, in which areas of fibrosis of various ages are interspersed with normal lung. This pattern could be explained by repeated episodes of lung injury followed by abnormal wound healing responses. The cause of the initiating alveolar epithelial injury is unknown, but postulated mechanisms include immunological, microbial, or chemical injury, including aspirated gastric refluxate. Reflux is promoted by low basal pressure in the lower oesophageal sphincter and frequent relaxations, potentiated by hiatus hernia or oesophageal dysmotility. In susceptible individuals, repeated microaspiration of gastric refluxate may contribute to the pathogenesis of IPF. Microaspiration of nonacid or gaseous refluxate is poorly detected by current tests for gastroesophageal reflux which were developed for investigating oesophageal symptoms. Further studies using pharyngeal pH probes, high-resolution impedance manometry, and measurement of pepsin in the lung should clarify the impact of reflux and microaspiration in the pathogenesis of IPF.

## 1. Introduction

Idiopathic pulmonary fibrosis (IPF) is the most common idiopathic interstitial pneumonia and carries a prognosis worse than many cancers. Despite the progressive and ultimately fatal nature of this disease, it remains poorly understood, and there are no effective disease modifying treatments. Classical hypotheses regarding the pathogenesis of IPF focused on a chronic inflammatory model leading to fibrosis, but the resulting treatment strategies focusing on anti-inflammatory agents have proven largely ineffective in altering the disease course and mortality [1–3]. Over recent years, our understanding of this condition has moved away from the inflammatory model towards a hypothesis focusing on alveolar epithelial injury followed by abnormal tissue repair and aberrant wound healing [4]. This model proposes that failure of normal re-epithelialisation following loss of alveolar-capillary basement membrane secondary to lung injury results in cytokine-mediated fibroblast proliferation and subsequent fibrosis. Therefore, it is suggested that development of this disease requires underlying susceptibility combined with exposure to a source of lung injury. Much work has been done exploring genetic susceptibility to IPF, prompted by the recognition of families affected by pulmonary fibrosis [5]. Numerous genetic polymorphisms have been studied including the major histocompatibility complexes [6], tumour necrosis factor-*α* [7], Fcgamma receptors [8, 9], and telomerase [10], and positive associations with IPF have been demonstrated. Short telomeres and telomerase mutations, which may compromise cell renewal capacity in tissues, have been demonstrated in peripheral blood leukocytes in some IPF families and in a subset of sporadic IPF cases [11]. These findings suggest that IPF may be a disorder of lung regeneration, and although none of these factors have been found to be either necessary or sufficient to cause the disease in isolation, pulmonary fibrosis may ensue only in response to certain stimuli. 

**Figure 1:**
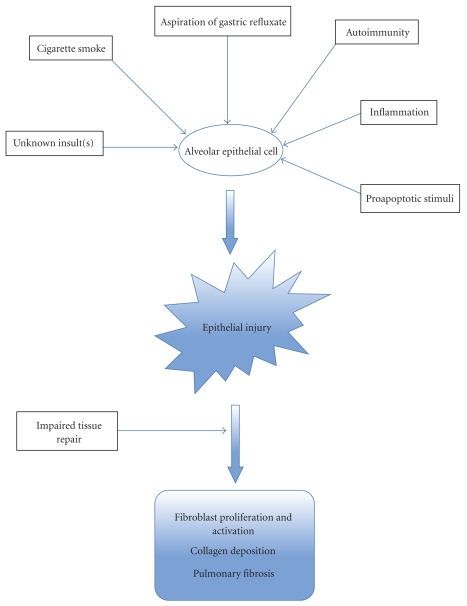
“Epithelial hypothesis”: proposed mechanisms of alveolar epithelial injury and fibrosis in IPF.

Understanding the source of initial lung injury is central to understanding IPF. Proposed injurious agents include viruses [12, 13], autoantibodies [14], and chemicals including the reflux and aspiration of acid or nonacid material from the gastrointestinal tract (Figure 1).

## 2. Pathophysiology of Abnormal Reflux

Abnormal Gastroesophageal reflux occurs when there is failure of one or more of the physiological protective mechanisms. The reflux of gastric contents in health is prevented through the combined actions of the oesophageal musculature including the lower oesophageal sphincter (LES) that must maintain a normal tone and frequency of transient relaxations and the diaphragmatic crura providing an extrinsic pressure. Phonation alters the anatomy of the crural diaphragm and immediately predisposes to reflux in humans. Disorders affecting the LES can be functional (increased frequency of transient relaxations) or mechanical (reduced LES tone) and can be caused by a number of factors including hiatus hernia, certain foods, and drugs. Cigarette smoking results in reversible relaxation of the lower oesophageal sphincter with an early study demonstrating that two-thirds of cigarettes smoked result in a reflux episode in symptomatic individuals [15, 16]. Cigarette smoking is associated with an increased risk of developing idiopathic pulmonary fibrosis, with a negative impact on prognosis, but the nature of this relationship and the role of reflux has not been explored [17]. 

An additional factor influencing Gastroesophageal reflux is the pressure gradient between the abdomen and the thorax. It is suggested that the increased negative intrathoracic pressure associated with diseases that reduce lung compliance may predispose to reflux [18, 19]. Gastric refluxate may be liquid, gaseous, or particulate; acid or nonacid; distal (localized to the distal oesophagus) or proximal (reaching the proximal oesophagus and pharynx) [20, 21]. Heartburn as a symptom of liquid acid reflux in the distal oesophagus is common in the general population [22, 23]. However, a significant proportion of Gastroesophageal reflux is asymptomatic [24] and may indeed be underestimated in the many studies that have used only pH monitoring, which fails to detect nonacid reflux. Potentially injurious agents in nonacid or weak-acid refluxate include bile salts and enzymes including pepsin. Studies of reflux in patients with laryngeal and respiratory symptoms have shown that the concept of reflux being synonymous with heartburn is outdated. Proximal reflux is strongly associated with laryngeal symptoms such as dysphonia, hoarseness, and throat clearing. Abnormal oesophageal peristalsis may be an important contributor to extra-esophageal symptoms of reflux by prolonging esophageal acid clearance time (i.e., refluxate remains in the proximal oesophagus for longer time) [25]. It is increasingly recognised that gaseous or particulate proximal acid or nonacid reflux is associated with a variety of respiratory conditions including chronic cough [26, 27], asthma, COPD, and bronchiectasis [28, 29]. Detection of pepsin and bile salts in bronchoalveolar lavage (BAL) fluid provides unequivocal evidence of aspiration of refluxate into the lower respiratory tract, which is referred to as microaspiration in the absence of a classical major clinical aspiration event [30]. The association of Gastroesophageal reflux with fibrotic lung disease has historical roots [31–33] although a precise temporal relationship between pulmonary fibrosis and reflux has not been established and there is uncertainty over cause or effect. However, the hypothesis that repeated microaspiration of aerosolised particles secondary to Gastroesophageal reflux leads to alveolar epithelial injury, and subsequent fibrosis is attractive and is worthy of further investigation.

## 3. Studies of Gastroesophageal Reflux and IPF

The notion of recurrent microaspiration as a potential cause of pulmonary fibrosis is an old one, with reported case series dating back over half a century [31], but clinical studies came later with the advent of oesophageal physiology technology. In 1979, Pellegrini and colleagues [34] published a study of 100 patients with reflux investigated by pH monitoring and found that patients with oesophageal hypomotility and weak peristalsis were more likely to have respiratory symptoms. There was a limited correlation between typical oesophageal reflux symptoms (e.g., heartburn) and objective reflux events. A number of studies have subsequently attempted to evaluate the exact prevalence of reflux in IPF (Table 1).

In a prospective study of 17 consecutive IPF patients, Tobin and colleagues [35] found a significantly higher prevalence of oesophageal acid reflux (detected by ambulatory pH monitoring) in the IPF group. Most patients with IPF and abnormal oesophageal acid exposure did not have typical oesophageal reflux symptoms such as heartburn or regurgitation. In this small study, there was no significant correlation between lung function (DLCO) and acid exposure times.

Raghu et al. [36] conducted the largest prospective study of IPF patients to date to determine the prevalence and characteristics of GER in this population. Sixty-five IPF patients were evaluated in this study and compared with 133 patients with intractable asthma. There was a significantly higher prevalence of reflux in the IPF group, but there was no significant correlation between the severity of IPF and the percentage of proximal and distal oesophageal acid reflux time. Only 47% of the IPF patients had typical reflux symptoms of heartburn or regurgitation. Furthermore, 65% of patients were taking a proton pump inhibitor at the time of the study suggesting that 87% prevalence of reflux might be an underestimate. On the basis of these findings, the authors suggested that investigation with oesophageal pH testing was indicated for IPF patients irrespective of symptoms of acid reflux. 

In a prospective study of 28 consecutive patients with IPF, Bandeira and colleagues [38] evaluated the prevalence of Gastroesophageal reflux disease (GERD) by oesophageal manometry and pH studies and divided the study population into GERD^+^ and GERD^−^ groups. In GERD^+^ group, 77% had heartburn or regurgitation in comparison to 33% in the GERD^−^ group, supporting the suggestion that oesophageal symptoms alone are inadequate markers of GER. In terms of oesophageal motility studies, the most common findings were of oesophageal hypomotility and LES hypotonia. There was no significant difference in clinical or functional characteristics (including FEV1, FVC, and DLCO) between the GERD^+^ and GERD^−^ groups.

The association of reflux with parenchymal lung disease has also been evaluated in connective tissue disease-associated pulmonary fibrosis, and in particular scleroderma. In a prospective study of 40 consecutive patients with scleroderma [39], there were significantly higher number of acid and nonacid reflux episodes in patients with interstitial lung disease compared to patients with normal thoracic high-resolution computed tomography (HRCT) scans. Furthermore, there was a good correlation between pulmonary fibrosis scores and reflux events at both the proximal and distal oesophagus. 

There is a high prevalence of Gastroesophageal reflux in IPF patients referred for lung transplantation. Sixty-seven percent of patients had Gastroesophageal reflux in a cohort of 30 patients in a study by Sweet and colleagues [40]. Moreover, 65% of those with reflux had a hypotensive LES. The presence of typical reflux symptoms of heartburn, regurgitation, or dysphagia had limited sensitivity and specificity for the detection of distal oesophageal reflux, supporting the suggestion that oesophageal symptoms are a poor screening tool to detect abnormal reflux in IPF patients, and the threshold to consider underlying reflux disease in this population should be low.

The role of reflux and microaspiration in the pathogenesis of lung disease is strengthened by a retrospective analysis of 457 patients undergoing lung transplantation [41]. The study population was stratified into four groups on the basis of history of typical reflux symptoms and timing of fundoplication. The group of patients who underwent fundoplication prior to lung transplant or shortly after transplant had a lower incidence of acute rejection/bronchiolitis obliterans syndrome (BOS) and better survival than the group which did not undergo antireflux surgery. This study has led to suggestions that microaspiration of stomach contents may play an important role in the pathophysiology of posttransplant BOS, and gastric refluxate could be an inciting trigger for transplant rejection.

These studies support the notion that GER is associated with IPF. However, GERD is common in the general population (10%–20% in the Western World) and up to 50 reflux events per 24 hours is considered normal, so it is not possible to draw a firm conclusion regarding a cause-and-effect relationship. Conceivably, reflux could be a secondary event in patients with IPF because of mechanical effects. Advanced fibrosis is associated with decreased lung compliance and increased negative pleural pressure, thus potentially promoting reflux of gastric contents into the oesophagus [19].

## 4. Pathophysiology and Histology of Lung Injury

We and others [42–44] have found that histopathological analysis may support a diagnosis of reflux and aspiration in selected cases of pulmonary parenchymal injury. A distinct histological pattern of “centrilobular fibrosis” has been described that is possibly associated with GER and aspiration [44]. This may be observed in patients with scleroderma, who are especially prone to GER and aspiration due to oesophageal involvement in their disease. The presence of foreign material in a lung biopsy specimen along with a pattern of centrilobular fibrosis is highly suggestive of aspiration-induced lung injury.

Another distinct clinicopathological entity associated with centrilobular and peribronchiolar fibrosis is bronchiolocentric interstitial pneumonia. Yousem and Dacic [45] reported 10 cases of this interstitial lung disease with patchy lymphocytic alveolitis without granuloma formation. It can be speculated that Gastroesophageal reflux may play a role in the development of this distinct interstitial lung disease as aspirated material from gastro and extra-esophageal reflux would have a propensity to cause interstitial pneumonia in a centrilobular and peribronchiolar distribution.

Whilst lower oesophageal liquid acid reflux leading to heartburn is associated with respiratory disease including IPF in epidemiological studies, it is more difficult to detect proximal gaseous reflux that is likely to be aspirated into the airways and alveoli. This type of reflux may elicit symptoms of hoarseness, throat clearing, cough, wheeze, and breathlessness, and the threshold for manifesting these symptoms depends on neural sensitivity of the larynx and airways [46]. The precise injurious agents in refluxate have not been characterized in detail but may include enzymes and bile salts. Nonetheless, the airways and alveoli would seem to be poorly designed to resist the noxious refluxate once it has traversed past the larynx. At present, we can only speculate about why some patients would respond to airway microaspiration with cough or wheeze, whereas in others, the refluxate is transported to the distal airspaces, where it may induce alveolar epithelial injury or apoptosis. These questions are worthy of further investigation.

## 5. Acute Exacerbations of IPF and Gastro-Esophageal Reflux

The natural history of IPF is variable. Some individuals exhibit periods of relatively stable lung function followed by acute deterioration in respiratory function with no obvious cause [47], which is referred to as an acute exacerbation of IPF (AE-IPF). A number of studies have reported this clinical entity which is associated with high mortality (20%–86%) [48–50]. The most common histological pattern seen in AE-IPF is diffuse alveolar damage superimposed on UIP pattern [51–53], although organizing pneumonia has also been reported [48]. The etiology of these exacerbations is unknown, and one of several hypotheses suggested for AE-IPF is Gastroesophageal reflux and aspiration [54].

## 6. Investigations and Treatment

A clinical suspicion of reflux and microaspiration should not rely on oesophageal symptoms of heartburn and regurgitation but will be increased if cough is a prominent symptom, especially if it occurs on phonation or is related to certain foods or meals or occurs on rising from bed (when the LES relaxes) [55]. Further investigations are often unhelpful—for example, upper gastrointestinal endoscopy is often grossly normal. Our practice with IPF patients is to institute an empiric trial of medical treatment if clinical suspicion of reflux is high, particularly if the patient reports a chronic cough. It should be accepted, however, that good quality clinical trial evidence is lacking, even in studies of patients with chronic cough in the absence of lung disease. In patients who report heartburn, we prescribe high-dose proton pump inhibitor therapy (e.g., lansoprazole 30 mg twice daily), and for patients without heartburn, we prescribe a gastric motility agent (e.g., domperidone 10 mg three times daily), in combination with dietary and behavioural advice. Ambulatory oesophageal pH monitoring fails to detect nonacid reflux, but concurrent impedance manometry can be used to investigate esophageal peristalsis. Some patients with reflux and microaspiration regurgitate from the proximal esophagus before the food reaches the stomach, and this predicts a poor response to antireflux therapy. Surgical fundoplication effectively treats acid and nonacid reflux in the majority of patients—its effectiveness in treating resistant heartburn or regurgitation is well proven, but its utility for extra-esophageal reflux symptoms is currently the focus of much investigation in a variety of settings [56]. Antireflux surgery should be considered in patients resistant to conventional medical therapy. There is evidence of a positive impact of antireflux surgery on exercise capacity and oxygen requirements in patients with IPF awaiting lung transplantation. Linden and colleagues [57] evaluated 149 patients on a lung transplant waiting list. Fourteen patients with IPF had significant reflux detected by oesophageal pH monitoring and symptom analysis despite being on medical therapy with proton pump inhibitors. These patients had a significant improvement in exercise capacity and need for supplementary oxygen following antireflux surgery. The laparoscopic route of fundoplication to treat gastroesophageal reflux has the advantage of reduced morbidity and shorter hospital stays compared with conventional open surgery. We believe the threshold for recommending this procedure in drug-resistant reflux manifesting as either oesophageal or extra-esophageal symptoms should be low in IPF patients.

## 7. Future Developments

The minimally invasive pharyngeal pH probe, which detects liquid as well as aerosolized acid, is inserted transnasally and rests just behind the soft palate for between 24 and 48 hours, and data is transmitted by wireless telemetry to a recorder (Restech Dx-pH measurement system, Respiratory Technology Corporation, San Diego, CA, USA). In our department, the pharyngeal probe has detected acid reflux in the upper airway in a series of patients in whom oesophageal pH monitoring failed to detect any significant Gastroesophageal acid reflux [58]. High-resolution impedance manometry is another new technique that should better characterize nonacid reflux. A series of 36 sensors distributed at 1 cm intervals along the length of the oesophageal probe permit simultaneous pressure measurements, particularly regarding the individual components of the antireflux barrier comprising the lower oesophageal sphincter and the diaphragmatic crura. Finally, detection of the gastric enzyme pepsin in exhaled breath, induced sputum, or BAL has been used successfully for investigating aspiration episodes. These techniques need to be applied in combination to better determine the temporal relationships between reflux and development and progression of IPF in a large population of patients.

## 8. Conclusion

There is a need for further investigation into the association of GER and IPF in an attempt to substantiate any causal link since GER could predispose to or directly incite alveolar epithelial injury leading to parenchymal fibrosis. It will be important to investigate nonacid and gaseous reflux in greater detail utilizing both subjective and objective measures.

##  Conflict of Interests

None of the authors report any potential conflict of interest.

## Figures and Tables

**Table 1:** Prominent clinical studies evaluating gastroesophageal reflux in IPF.

Study	Methodology	Number of subjects	Prevalence of GERD	Other outcomes
Tobin et al. 1998 [35]	Prospective with non-IPF ILD control	17 IPF8 controls	94% IPF50% controls	25% of IPF patients had typical reflux symptoms
Raghu et al. 2006 [36]	Prospective, control group without ILD	65 IPF133 asthmatics	87% IPF68% Asthma	47% of IPF patients had heartburn and regurgitation. No significant difference in proximal reflux in IPF and asthma, 63% versus 61%, respectively
Raghu et al. 2006 [19]	Retrospective case review	4 IPF	100% as one of the inclusion criteria	2–6 year follow up with stable FVC and TLCO with proton pump inhibitors
Salvioli et al. 2006 [37]	Prospective	18 IPF10 secondarypulmonary fibrosis	67% of IPF patients had abnormal distal reflux	57% of total patients had heartburn and regurgitation
Bandiera et al. 2009 [38]	Prospective	28 IPF	35.7%	Participants divided into GRED^+^ and GERD^−^ groups

## Abbreviations

GER: Gastroesophageal reflux
GERD: Gastroesophageal reflux disease
HRCT: High-resolution-computed tomography
IPF: Idiopathic pulmonary fibrosis
ILD: Interstitial lung disease
LES: Lower oesophageal sphincter
BAL: Bronchoalveolar lavage.
